# Leaky Pipeline Myths: In Search of Gender Effects on the Job Market and Early Career Publishing in Philosophy

**DOI:** 10.3389/fpsyg.2017.00953

**Published:** 2017-06-14

**Authors:** Sean Allen-Hermanson

**Affiliations:** Department of Philosophy, Florida International University, MiamiFL, United States

**Keywords:** underrepresentation, gender bias, sexism, hiring, philosophy

## Abstract

That philosophy is an outlier in the humanities when it comes to the underrepresentation of women has been the occasion for much discussion about possible effects of subtle forms of prejudice, including implicit bias and stereotype threat. While these ideas have become familiar to the philosophical community, there has only recently been a surge of interest in acquiring field-specific data. This paper adds to quantitative findings bearing on hypotheses about the effects of unconscious prejudice on two important stages along career pathways: tenure-track hiring and early career publishing.

## Introduction

That philosophy is an outlier in the humanities when it comes to the underrepresentation of women has been the occasion for a lot of discussion about possible effects of subtle forms of prejudice, including implicit bias and stereotype threat. Though real-world effects are not strongly evidenced (Forscher et al., 2017), there is widespread concern in philosophy that involuntary and unconscious implicit associations might diverge from a person’s declared beliefs affecting our actions, judgments, and attitudes. Unconscious bias might influence how we treat junior colleagues from socially stigmatized groups when it comes to sharing opportunities for professional development, advancement, and in evaluating scholarly potential and credentials. For example, a departmental committee might implicitly prefer a male candidate to a female candidate with the same qualifications despite holding conscious and explicit attitudes about the equality of the sexes. Meanwhile, stereotype threat is when awareness of and identification with stereotypes (such as that philosophy is for white males) results in heightened anxiety, performance disparities, and reduced interest.

These ideas have become familiar to the philosophical community, which continues to debate policy initiatives and other measures for improving diversity, such as making syllabi and conference line-ups more inclusive, adjusting the management of professional organizations, and reforming journal and hiring practices. These ongoing discussions need to be informed by the best possible evidence, and there is a growing interest in acquiring field-specific data. The investigatory model informing this study is inspired by the hiring audits used in STEM disciplines. This paper contributes data pertinent to hypotheses about the effects of prejudice on two important stages of career pathways: tenure-track hiring and early career publishing.

If women are evaluated more harshly because of unconscious bias on the part of letter writers and hiring committees, or have weaker files and perform less well in interviews because of stereotype threat, or even face conscious and explicit discrimination, then they might be expected to be less successful at finding tenure-track employment. Indeed, biases are often conjectured to be a major cause of the underrepresentation of women in philosophy.^1^ Fortunately there have been several recent studies of employment trends, including Jennings’ for a 2-year period (2012 and 2013) and a follow-up funded by the American Philosophical Association known as the APDA report.^2^ These and other resources can help test hypotheses predicting effects of biases on hiring.^3^ Working from her original data, it was decided that post-doctorate appointments would be ignored in order to focus on more desirable tenure-track lines, leaving us with 229 men and 109 women in the pool. Since this manuscript was written, the data used in the APDA report has been corrected and revised and will also be taken into account. So, how are women doing on the job market?^4^.

## Results

Analysis of Jennings’ original data suggests women and men are hired at a rate roughly proportionate to their numbers for entry-level tenure-track jobs in philosophy.^5^ Concerning the follow-ups, the first APDA report in 2015 actually found women were hired significantly more often, increasing the odds of obtaining a permanent academic position by 85%.^6^ A 2016 update corroborated this finding notwithstanding Jennings’ statement that “we did not find a significant effect of gender on placement…”^7^ While technically true, however, this was only because in 2016 they elected not to examine this low hanging fruit. Despite this flagging of interest in this aspect of the gender question, she did acknowledge there were about 10% more women than men among those obtaining a permanent position over the entire data set (chi-squared test *p* < 0.01) and (with prompting by an anonymous commenter) admitted the number was much higher (around 23%) concerning those graduating within the most recent hiring cycles (2012–2015). Another commentator also brought the high significance of this result to our attention (chi-square, *p* = 0.0007). The upshot is that with the help of various anonymous commentators, we can be confident that the APDA findings offer strong support that men are significantly less likely to obtain permanent positions.

**FIGURE 1:**
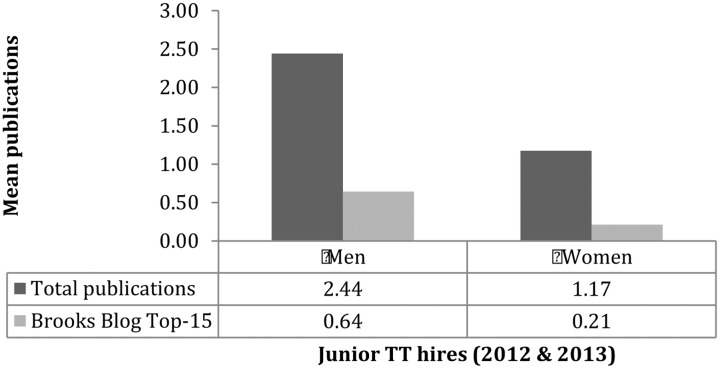
Gender and publishing.

Another noteworthy finding obtained from Jennings’ earlier results is that female candidates had about half as many publications as their male counterparts. The average publication counts for candidates (having no prior academic appointment) were 1.37 for men and.77 for women (medians 1 and 0; *p* = 0.000808)^8^ and is also extremely significant. However, as the quantity of publications is only one very crude measure of a candidate’s strengths, we can also look at how several other variables might depart from these aggregated results. For example, besides quantity, Jennings’ data also contained information about the putative “quality” of a publication (defined as a “top-15” journal according to a poll at the Brooks Blog). Here, male tenure-track hires are about three times more likely to have published in a highly regarded venue (see Appendix and **Figure 1**).

It is natural to wonder how important publications are when it comes to assessing job candidates, and certainly we can agree publications are not the only relevant factor. Even for a research-oriented position the quality of writing samples, the reputation of doctoral institutions, and the weight assigned to letters of recommendation will also be taken into account. As a rough proxy for reputational factors rankings of degree-granting programs obtained from the 2006–2008 edition of the Gourmet Report were utilized.^9^ How publication records of new hires might differ depending on whether they had a prior position or accepted a tenure-track job straight out of graduate school was also considered.

Some have claimed that prestige interacts with gender in that women from highly regarded programs tend to publish the least, whereas men from less fancy programs publish the most.^10^ However, we find the relationship between gender and prestige to be somewhat murkier. Although the finding men in general tend to publish more and in more prestigious places was corroborated, program prestige correlated positively with the output of high-quality publications regardless of gender. Gendered differences also depended somewhat on whether we were looking at candidates who had held a prior academic appointment.

**FIGURE 2:**
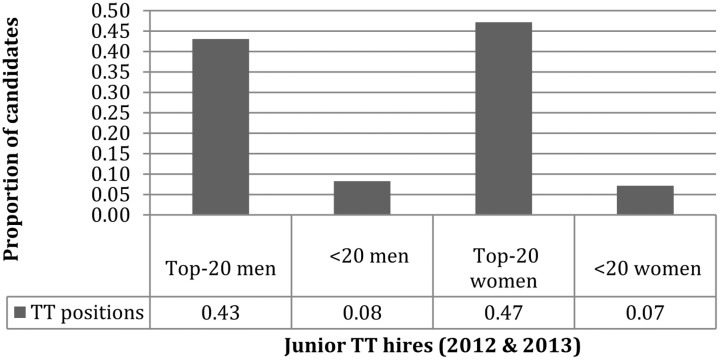
Gourmet-ranked TT positions.

First, mean Gourmet Report scores were incorporated within Jennings’ spreadsheet revealing a disparity in average home department rankings of 3.31 for men and 2.93 for women (medians were 3.6 and 3.2). High prestige “top-20” departments have a score of at least 3.4, and so next men and women were divided into elite (“top-20”) and non-elite (“non-top-20”) subgroups to see if there would be any interesting effects.^11^ The rankings for male and female “top-20” hires turn out to be very similar with mean scores of 3.94 for men and 4.02 for women (with medians of 3.7 each). Given this, we expect to find a small gendered difference in prestige among the remainder, and indeed the averages here are 2.26 for men and 1.9 for women (with medians of 2.6 and 2.3).^12^ It was also noted that there was no significant interaction between gender and the prestige of hiring departments, though there was evidence candidates from relatively lower prestige institutions lack upward mobility: Whereas top candidates of either gender could expect to find a position at a Gourmet-ranked institution a bit less than half the time, this was true of other candidates only 7–8 percent of the time (**Figure 2**). This might indicate that there are, in effect, two semi-independent job markets. In terms of outcomes, there seems to be a top-20 market mostly closed to non-elite candidates and a non-top-20 market open to all.

**FIGURE 3:**
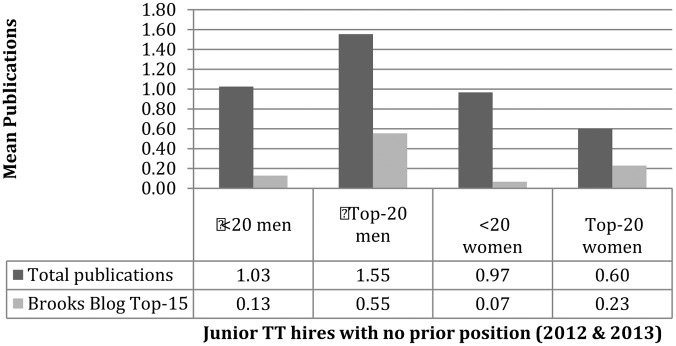
Publishing (no prior position).

**FIGURE 4:**
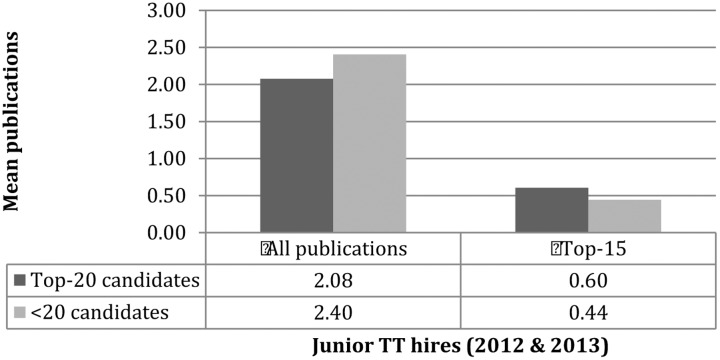
Prestige and publishing.

Next, turn to consider how prestige might interact with gender when it comes to publishing. As mentioned earlier, differences depended on whether candidates had a prior appointment. In considering those with no prior appointment, it was found that top-20 men stand out: they publish more, and in “better” places than the others. Meanwhile, top-20 women publish less often than non-top-20 men and women, nevertheless they tend to do better when it comes to quality (**Figure 3**). As it is unclear how to weight quantity versus “quality” in assessing candidate strength, no conclusions are drawn here about the advantages or disadvantages of the remaining subgroups. We can observe that top-20 women have much more access to top-20 jobs, which might suggest “quality” counts for more across the market. Alternatively, there might be different standards for the different “markets” proposed above: top-20 individuals appear to be a little stronger concerning “quality” and non-top-20 are stronger for quantity (**Figure 4**). Perhaps then publishing counts, but counts differently depending only whether one is competing on the “elite” market favoring “quality” or the “non-elite” market favoring raw output.^13^

**FIGURE 5:**
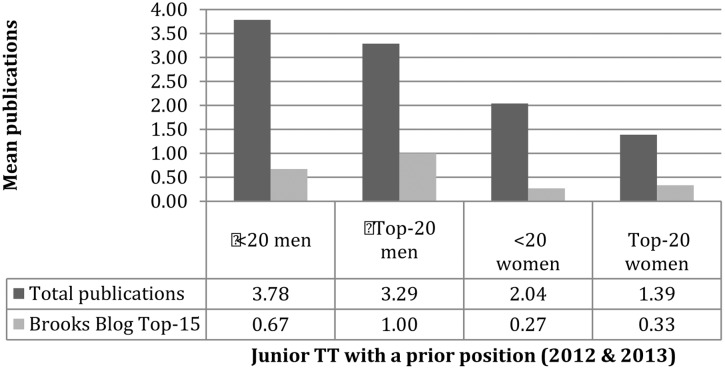
Publishing (with prior position).

Now we can consider the candidates who did have a prior position. Here, men had significantly higher averages for quantity and “quality” (**Figure 5**). For example, low-prestige men published almost three times as much as the average high-prestige women and were about two times more likely to have a top-15 publication. This might indicate that women, regardless of prestige, tend to submit to journals less frequently because they are less confident, as expected by hypotheses invoking stereotype threat^14^ or even disadvantages in the reviewing process.^15^ Then again, there are several other explanations for the publishing gap. Notwithstanding this uncertainty, men and women appear to be held to different standards.

Returning to an earlier suggestion, might it be the case that publications are not that important in hiring? This is hard to accept given that productivity is so often tied to securing research-intensive positions in the competitive academic environment and critical to determinations about prospects for earning tenure. This seems clear when we consider lateral hires, which constitute much of the data, and as just mentioned indicate upward trends in output. That candidates from less fancy programs publish more regardless of gender also suggests a widespread presumption that publishing compensates for other deficiencies. We can also note that previous research indicates that publication records are a critical indicator of candidate strength (Steinpreis et al., 1999).

In a blog comment, Jennings^16^ attempts to explain away the publishing gap by proposing that enhanced opportunities are more often offered to males. Others have also worried that “at the graduate level, supervisors may be more likely to encourage men to publish their work” (Saul, 2013). Jennings wonders if most of the difference between genders is attributable to a handful (15%) of high productivity (HP) men, defined as those with at least five publications at the time of hiring (5% of women are HP by the same standard).^17^ Yet these numbers are derived from looking at all hires, including those candidates who had a prior academic appointment, and therefore more opportunities to publish. When we consider those with no prior position, none of women and only 3% of the men (*n* = 7) are HP. Since there are so few, the gap cannot be explained by those who are highly productive.

Nevertheless, all HP hires were examined in order to see what proportion of their work might be attributable to enhanced opportunities.^18^ Using Google searches of cvs the number of such publications for each of the 61 HP candidates was obtained by counting works that were co-authored with a senior figure, articles or chapters in edited volumes, conference proceedings, and publications which otherwise appeared to be by invitation, such as introductions to special issues.^19^ Although the pool of HP women is small^20^ it was found that 37.5% of their publications fell into this category. Turning to the men, first those who were exceptionally productive (having at least 10 publications) were examined. The rationale here is that if the HP men are favored with extra opportunities, this will likely be reflected in the output of those who publish the most. Yet for this group, only 34.3% of their work was attributable to enhanced opportunities^21^ while the result was 37.7% for all HP men.^22^ In addition, the means, medians, and modes for those in the HP group did not significantly vary by gender. While not exhaustive, as there could be other kinds of special opportunities, favoritism in a non-blind review process, as well as differential barriers to obtaining prior positions, the data offered here suggests HP men and women are treated about equally. It was found that highly productive men were about twice as likely to publish in well-regarded (“top-15”) journals.^23^

Returning to market outcomes, the previous results were augmented by placement data obtained from two additional sources: the American Philosophical Association’s Guide to Graduate Programs^24^ and the Philjobs website^25^ and cohere with similar findings from hiring audits in STEM fields (Williams and Ceci, 2015). In addition, individual notices of new appointments from Philjobs for the 2014 hiring season were monitored.^26^ Next the analysis of the APA data is presented followed by a consideration of the findings obtained from Philjobs.

Data was transcribed about gender and hiring found in the APA’s 2013 and 2014 Guides to Graduate Programs for two 5-year periods: 2008–2013 and 2009–2014. Only programs that allowed for a comparison between hiring outcomes and how many men and women went to market were included in the calculations. The Guides provided data for 64 schools in the 2013 edition and 65 for 2014 (37 schools provided data twice, so there is placement information available for 92 distinct programs for these mostly overlapping timespans). For 2008–2013 it was found that 40% of men who went on the market eventually landed a tenure-track job compared to 50.6% of the women, meaning a woman’s probability of obtaining tenure-track employment was about 25% better (*p* = 0.037).^27^ The corresponding probabilities of obtaining any kind of academic position (including much less desirable temporary appointments) were a lot closer at 86 and 89%. Women also made up 26.3% of the market and 31.1% of the tenure-track placements. The 2014 Guide reinforces this pattern, with 35.3% of men and 46.7% of women finding tenure-track employment from 2009 to 2014 meaning the probabilities were about one-third better for women (*p* = 0.016).^28^ Similar to before, 83.6% of men and 87.8% of women found any kind of academic job. Women made up 25.2% of the market and 31% of junior tenure-track hires.

One might wonder if schools with good placement records, and, especially, good records for placing women might be overrepresented in the APA data. However, this concern is not realistic. Consider what it would take for the schools where we didn’t have data to close the gender gap. According to the APA, the 2008–2013 period comprised 530 junior tenure-track placements, and yet we would have to suppose an additional 200 competitions went unreported in which a man won every single time—in that case the chances equalize to 50%. There would have to be more than 500 unreported tenure-earning jobs going solely to men for the disproportion to be reversed (i.e., for men to have a 25% greater chance). In such a small profession, there are probably not enough unreported jobs for this to be the case: while Philjobs reported 816 junior tenure-track placements for the same period, many of these are lateral moves that placement officers would not normally pass on to the APA.

Adding to uncertainty about possible unreported hires, one might also wonder if these results would hold up for periods other than 2009–2014, and what the year-to-year results look like. With these concerns in mind we can turn to data provided by Philjobs. This process began by examining the 2014 hiring season, which was arbitrarily defined as spanning July 1, 2014 to June 30, 2015. Over the course of the year information was gathered about individual tenure-track hires, including those who had a previous academic appointment as well as those going to market straight from graduate school. For 2014 it was found that 56 out of 148 hires (37.8%) went to women. While the number of doctorates awarded to women as a percentage of the total doctorates in philosophy fluctuates somewhat from 1 year to the next, it was assumed that the year immediately prior would give a reasonable approximation of how gender is distributed on the job market; in 2013, for example, 27% were awarded to women.^29^

**FIGURE 6:**
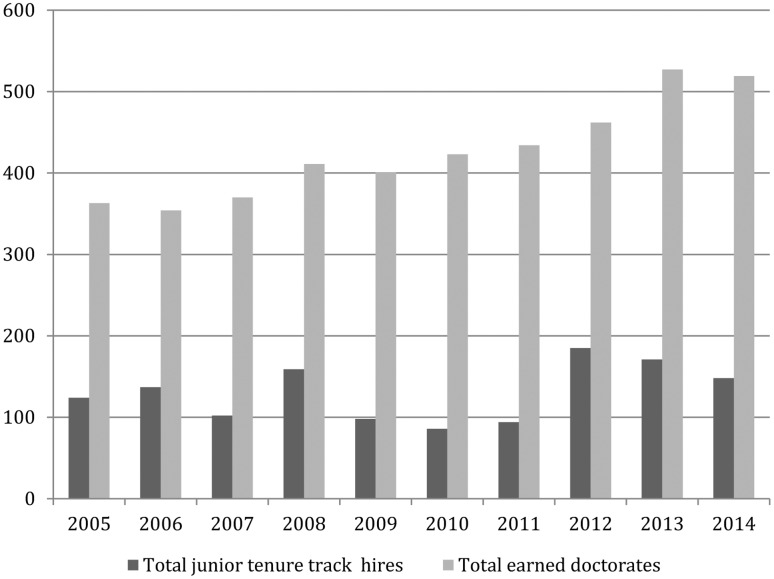
Earned PhDs and TT hiring.

**FIGURE 7:**
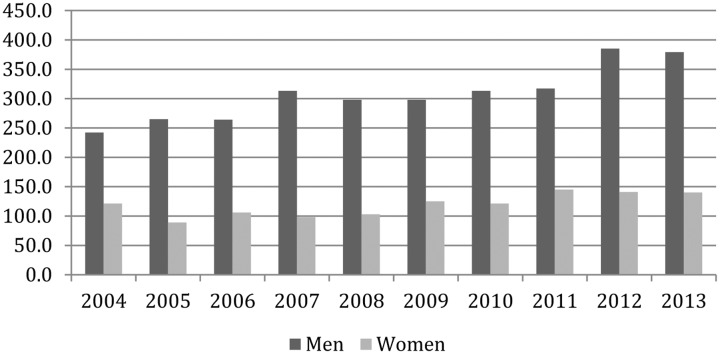
Earned Doctorates by gender.

**FIGURE 8:**
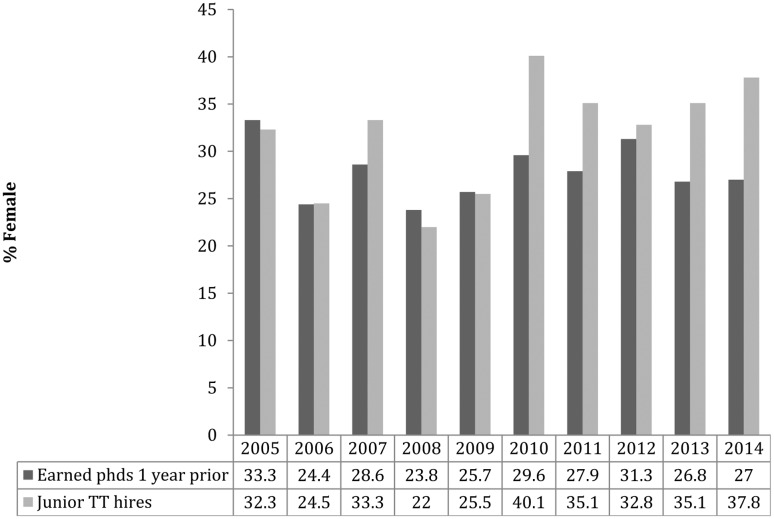
Gender and tenure-track hiring.

To add more depth to the investigation hiring outcomes for nine further years (2005–2013) were also examined using data provided by the Philjobs website. In order to make this information useable certain corrections and additions to their spreadsheet were necessary, including the elimination of duplicated entries, filtering out senior appointments and non-tenure-track hires, spot-checking for accuracy, and using Google searches to ascertain gender where it was missing or in doubt. Next, year-by-year comparisons were made between placement and the distribution of philosophy PhDs using the NSF’s Survey of Earned Doctorates. The relationship between awarded PhDs and junior hiring from 2005 to 2014 is depicted in **Figure 6** and gives a general sense of the market. The distribution of philosophy doctorates by gender for the same period is found in **Figure 7**. For some years (2005, 2006, 2008, 2009, 2012) there was a rough correspondence between the percentage of women who were hired and their share of philosophy doctorates awarded in the year immediately prior. As a further check the rankings for the 2005 market (using the 2001 edition of the Gourmet Report) were consulted, though there were no significant differences in the means (3.00 for men and 3.01 for women) or medians (men: 3.3; women: 3.35) of candidates. For the remaining years (including most recent hiring seasons) women appear to be overrepresented, accounting for 28.4% of the earned doctorates but 35.73% of tenure-track hires (**Figure 8**).^30^

Finally, these results were compared to updates found in the 2016 APDA report. First, my list of successful job candidates for the 2012 season was merged with the APDA’s. Although these mostly overlapped, there were some differences. In order to seek greater accuracy every candidate was re-checked, one-by-one, in attempts to verify gender and success in a tenure-track competition in 2012 (e.g., by consulting cvs, locating welcome messages at hiring Departments, etc.). Both data sets contained errors resulting in 56 changes to my list (37 additions and 19 deletions) and 36 changes to the APDA’s (29 additions and 7 deletions), thus bringing the two into harmony.^31^ Though this process was tedious and time-consuming, it was hoped it would maximize the accuracy of the data for at least 1 year and so allow us to see if this additional scrutiny would alter the results in any significant way. With this revised data it was then a simple task to recalculate the hiring figures. According to my original survey 32.7% of tenure-track hires went to women in 2012 whereas the APDA’s 2016 report puts this a little lower at 30.7%. The outcome for the revised and re-verified data is just shy of their result at 30.3%. To place this in context, note that in the previous year 31.3% of doctorates in philosophy went to women. Hence, it can be reaffirmed that the 2012 market outcomes do not attest to a significant gender effect in hiring. However, 2012 was also unusual in light of the pattern for the years 2010, 2011, 2013, and 2014, which might indicate significant bias in favor of female candidates (**Figure 8**). Would this pattern also stand up to further scrutiny? This time instead of more forensic checking of merged data sets, the APDA’s numbers were taken at face value with a result in keeping with my original findings provided in **Figure 8**. Going by the APDA’s data women obtained 32.5% of the tenure-track jobs in 2013 and 39% in 2014 whereas my results were 35.1% (with 26.7% earning doctorates) and 37.8% (27% earning doctorates). Instead of quibbling about a percentage point here or there, it can be agreed there is no evidence women are underrepresented among those obtaining tenure-track jobs for at least a decade. To the contrary, recent years seem to attest to a reverse gender effect.

## Discussion

Market outcomes starting in 2014 and going back 10 years offer no evidence women are at a disadvantage in tenure-track competitions. The same can be said for the other objective measures that were examined including publishing and the reputations of home and hiring departments. No statistically significant evidence that pervasive dysfunction in departmental cultures is harming early career market outcomes of budding women philosophers could be found. Meanwhile, the biggest drop in women’s participation appears to occur almost immediately, right after first exposure to philosophy’s themes, methods, and traditions (Adelberg et al., 2013; Dougherty et al., 2015). Although evidence that the gender gap in philosophy is attributable to pre-university influences has been available since at least 2012 (Paxton et al., 2012) the present study adds to the case against the hypothesis that sexist attitudes (whether conscious or unconscious) held by philosophers are a major cause of disproportion according to gender.

All the same, we can be somewhat reticent to draw strong conclusions about the extent of philosophy’s climate problems, and it might be premature to say that there is no systemic anti-female prejudice. Bias that was present but somehow neutralized by measures departments have taken or coping strategies adopted by women might have been overlooked. Then again it seems doubtful that explicit policy changes and coping strategies were adopted more than 10 years ago, long before there was wider awareness of the issue of unconscious bias. It is also conceivable that bias shows up elsewhere, affecting outcomes for tenure and promotion, though keep in mind this conjecture is not supported even by mainstays of the implicit bias literature, such as Steinpreis et al. (1999) whose name-swapping experiments found “no main effects” for tenurability. The present findings are a better fit with the strong preference for women in STEM found by experimental manipulations (Williams and Ceci, 2015).

While counter thoughts are not to be dismissed lightly, the hypothesis that unconscious bias works against women in hiring and early career publishing is not well supported. Although it is conceivable implicit bias initially reduces perception of a woman’s cv. and then “affirmative actors” reverse its impact, this proposal strikes one as overly complicated: why not just assume people are not downgrading the accomplishments of talented women?

The suggestion that there is a shyness effect making bias hard to detect is also hard to square with the evidence about pre-market publishing opportunities. Why doesn’t bias reveal itself in disparities for special invitations to publish where there are no equity policies or structures, little to no collegial oversight, and it is hard to conceive of coping strategies? We should also worry that efforts to improve the representation of women could even backfire, e.g., if committees adopted blind review of candidates under the dubious assumption that more accomplished women are systematically undervalued.

## Author Contributions

The author confirms being the sole contributor of this work and approved it for publication.

## Conflict of Interest Statement

The author declares that the research was conducted in the absence of any commercial or financial relationships that could be construed as a potential conflict of interest.

## Footnotes

1 “Implicit bias and stereotype threat…will make it harder for women to do well…to be recognized in graduate school, less likely to get strong letters of recommendation, and less likely to be hired. The women who, despite this, get hired at strong research departments are likely to be especially exceptional philosophers” (Saul, 2012).

2 The Academic Placement and Data Analysis project’s results can be found here: http://webcache.googleusercontent.com/search?q=cache:szvEA0v8tSgJ:dailynous.com/wp-content/uploads/2016/04/apdareportupdateto2015report.pdf+&cd=2&hl=en&ct=clnk&gl=us&client=safari

3 http://www.newappsblog.com/2014/12/gender-and-publications.html#more. See also Solomon and Clarke (2009).

4 Here and there minor corrections were made for errors, such as duplicate entries.

5 All of our raw data can be accessed at Genderandphilosophy.blogspot.com

6 See p. 11 of the 2015 report: http://dailynous.com/wp-content/uploads/2016/04/apdafinalreport2015.pdf

7 http://dailynous.com/2016/04/15/philosophy-placement-data-and-analysis-an-update/

8 Meaning a 99.9192% confidence that the result is not due to chance. *P* was obtained using a Fisher exact test.

9 It was reasoned that the reputational ranking should reflect the fact that candidates take around 5 or 6 years to obtain their degrees. But the results do not differ much if a slightly more recent or less recent edition is used instead.

10 See http://genderandprestige.blogspot.com

11 Note that candidates from top-20 schools appear to fill half of new tenure-line positions.

12 Women seem to be slightly more likely to obtain degrees from unranked programs, which were scored as a “0.” Hence, if we ignore unranked programs these small differences in the overall rankings disappear.

13 Against this the reader is asked to compare the data in **Figures 3**, **5**, which might suggest that when it comes to re-entering the market with a prior position it may be more important for non-elite candidates of either gender to improve “quality” and elite candidates to improve quantity.

14 Though we note that enthusiasm for stereotype threat theory is in rapid decline thanks to concerns about ecological validity, experimental design and interpretation, replicability, and evidence of publication bias, see Sesardic and De Clercq (2014).

15 This finding of a gender gap in publishing is in step with Krishnamurthy et al. (forthcoming).

16 http://www.newappsblog.com/2014/12/gender-and-publications.html#more

17 HP candidates were very likely to have had at least one prior position (median for both genders = 1; means were 0.88 for men and 0.86 for women).

18 In this case, we also included 10 highly productive individuals with post-doctorates in order to increase the population. *M* = 54; *F* = 7. Post-docs are shaded in blue in the spreadsheet titled “high performers” and tenure-track hires are in yellow.

19 We didn’t count book reviews as peer-reviewed publications. If we do, the proportion of work attributable to enhanced opportunities falls to 31%.

20 There are about a third as many as one would expect given that women account for 32.2% of the hires.

21 Falling to 31% with book reviews.

22 Falling to 32.8% with book reviews.

23 Using Jennings’ data, we found that the highly productive men (with at least five publications) had an average of 1.98 publications in top journals. Women had an average of 1.14. The median was 1 in both cases.

24 http://www.apaonline.org/?page=gradguide

25 http://philjobs.org/appointments/dataFeed

26 Philjobs http://philjobs.org/appointments

27 Calculated using a Fisher Test.

28 Fisher Test.

29 See the 2013 edition of the NSF’s Survey of Earned Doctorates.

30 *p* = 0.000372.

31 The final tally for 2012 was 140 men and 61 women. Note that there was only one instance where a gender was assigned incorrectly due to a limitation of the software used by the APDA project.

## APPENDIX

Raw data is available at genderandphilosophy.blogspot.com
